# A Pocket Text-Book of Diseases of the Eye, Ear, Nose and Throat, for Students and Practitioners

**Published:** 1901-03

**Authors:** 


A Pocket Text-Book of Diseases of the Eye, Ear, Nose and Throat, for Students and Practitioners. By William L. Ballenger, M. D., Assistant Professor of Otology, Rhinology and Laryngology in the College of Physicians and Surgeons, Chicago, etc., and A. G. Wippern, M. D., Professor of Ophthalmology and Otology in the Chicago Eye, Ear, Nose and Throat College. Duodecimo, 525 pages, with 150 engravings and six full-page colored plates Philadelphia and New York : Lea Brothers & Co. 1900. [Price, cloth, $2.00 net; flexible red leather, $2.50 net.]

This is a practical work, treating of diseases of the eye, ear, nose and throat. It is abundantly illustrated, possessing advantages for students and general practitioners, as diseases of these organs are among the most common conditions which medical men are called upon to handle.

The book reflects the most advanced state of its subjects, both in theory and practice, in diagnosis and treatment. The nomenclature of diseases of the eye is much less complex than in the ordinary textbook, while those diseases of the organ which the general practitioner is expected to deal with, are fully described.

In the section on the ear the physiologic tests of hearing are given considerable prominence. Suppurative diseases of the middle-ear and mastoid are fully described. The mastoid operation is dwelt upon with marked detail, each step being given which will interest the experienced aurist.

Obstructed nasal respiration is fully described in its etiological relation to many nasal and naso-pharyngeal diseases. The symptoms of suppurative diseases of the accessory sinuses of the nose are explained from their anatomical relations to each other, and to the turbinated bones. The section on diseases of the throat is concise but not exhaustive.—W. S. R.

